# Supplementary material for: MS2Lipid: A Lipid Subclass Prediction Program Using Machine Learning and Curated Tandem Mass Spectral Data
Source: Metabolites. 2024 Nov 7;14(11):602. doi: 10.3390/metabo14110602 (PMC11596251; doi:10.3390/metabo14110602)
Supplement: Supplementary file 1 [file metabolites-14-00602-s001.zip › Supplementary Files/Supporting Information File.pdf]

Supporting Information

MS2Lipid: a lipid subclass prediction program using machine learning and curated tandem mass spectral data

Nami Sakamoto[1], Takaki Oka[1], Yuki Matsuzawa[1], Kozo Nishida[1], Jayashankar Jayaprakash[2], Aya Hori[3], Makoto Arita[3,4,5,6,*], Hiroshi Tsugawa[1,3,5,*]

1. Department of Biotechnology and Life Science, Tokyo University of Agriculture and Technology 2-24-16 Naka-cho, Koganei-shi, Tokyo, 184-8588, Japan
2. Graduate School of Global Food Resources, Hokkaido University, Kita-9, Nishi-9, Kita-Ku, Sapporo 060-0809, Japan
3. Laboratory for Metabolomics, RIKEN Center for Integrative Medical Sciences, 1-7-22 Suehiro-cho, Tsurumi-ku, Yokohama, Kanagawa 230-0045, Japan
4. Division of Physiological Chemistry and Metabolism, Graduate School of Pharmaceutical Sciences, Keio University, 1-5-30 Shibakoen, Minato-ku, Tokyo 105-8512, Japan
5. Molecular and Cellular Epigenetics Laboratory, Graduate School of Medical Life Science, Yokohama City University, Tsurumi-ku, Yokohama, Kanagawa 230-0045, Japan
6. Human Biology-Microbiome-Quantum Research Center (WPI-Bio2Q), Keio University, 35 Shinanomachi, Tokyo 160-8512, Japan

Corresponding author

Hiroshi Tsugawa: htsugawa@go.tuat.ac.jp
Makoto Arita marita@keio.jp

Contents

Figure S1-4
Table S1–9
Supplementary Data S1–S4
Supplementary Note S1

Supplementary Figures

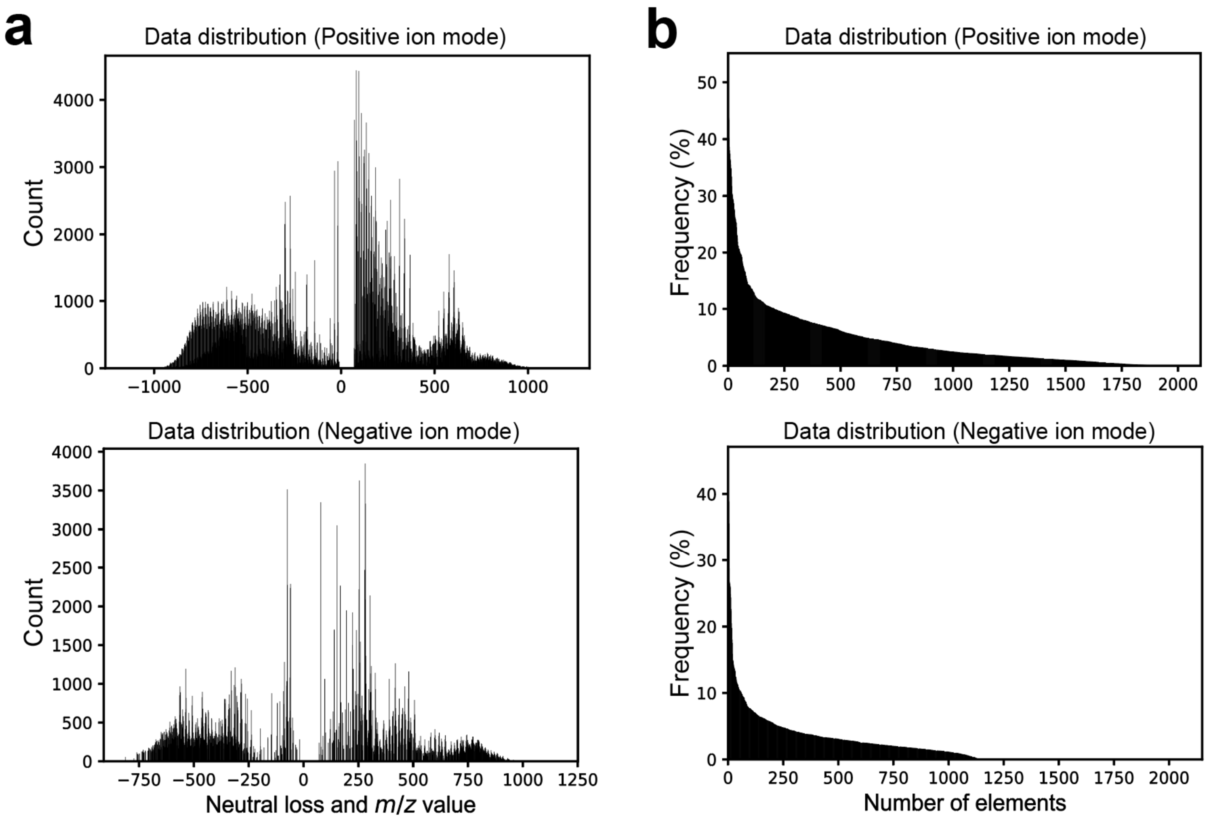

Figure S1. Frequency distribution of product ion and neutral loss features included in the spectral records. (a) Counts of product ion and neutral loss features (range: −1250 to 1250). The X-axis shows the *m/z* values of product ions and neutral loss values; product ions are displayed in the range of 0 to 1250, and neutral losses are displayed in the range of −1250 to 0. If an ion of *m/z* 100 is observed in a spectral record, a count of 1 is added to that feature. (b) Occurrence probability of product ion and neutral loss features. The Y-axis indicates the occurrence probability of each feature. The X-axis arranges the product ions or neutral losses in order of descending occurrence probability.

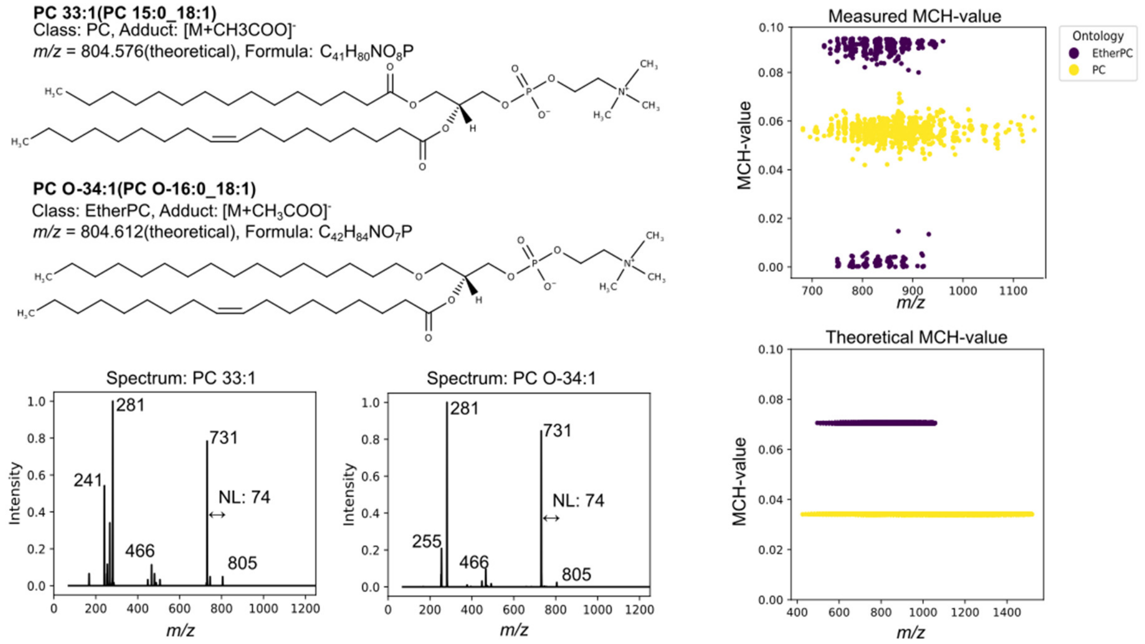

Figure S2. An example of MS/MS spectra and their MCH-values. Classification of MCH values based on PC and EtherPC structural formulas, MS/MS spectra, and oxygen atom counts in negative ion mode. The chemical structures and MS/MS spectra of PC 15:0_18:1 and PC O-16:0_18:1 were described. The MCH values of PC and ether PC included in our training set were calculated for the experimental (top-right panel)- and theoretical precursor (bottom-right panel) *m/z* values, respectively.

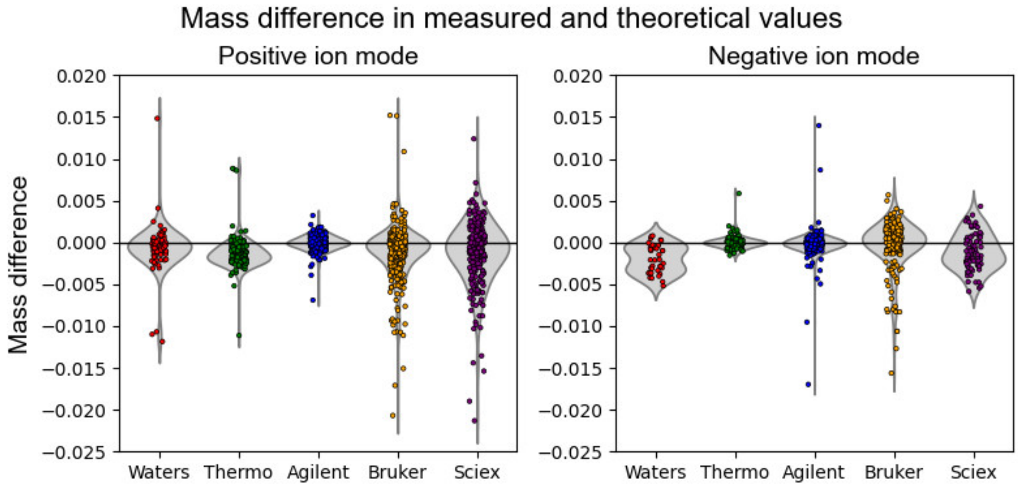

Figure S3. Mass difference between experimental- and theoretical precursor *m/z* values. The annotated peak features in RIKEN lipidomics database were used. The difference between the experimental- and theoretical *m/z* value was calculated, and the distribution was described for positive (left) and negative (right) mode data.

Spectrum labeled as “mix of PC and PE”

MS2Lipid result: 95.7% PE and 2.2% PC

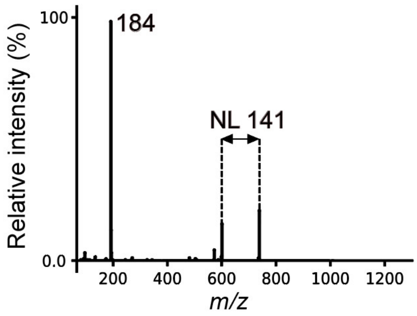

PC 33:2; [M+H]$^+$; *m/z* 744.554
Diagnostic ion: *m/z* 184

PE 36:2; [M+H]$^+$; *m/z* 744.554
Diagnostic ion: neutral loss of 141

Figure S4. Evaluation of scalability of MS2Lipid. MS2Lipid result for predicting the mixed spectra of phosphatidylcholine (PC) and phosphatidylethanolamine (PE).

Supplementary Tables

Table S1. MCH-values for lipid subclasses supported in MS-DIAL. The MCH-value was inserted into the original supplementary table provided in the previous paper (https://www.nature.com/articles/s41587-020-0531-2).

Table S2. Definitions of ClassyFire class and LIPIDMAPS/MS-DIAL lipid subclass

Table S3. Parameter settings for MS-DIAL analyses

Table S4. Retention times of metabolites used for retention time corrections

Table S5. Prediction results from ms2lipid in the test data

Table S6. The number of spectral records used in this study, MassBank, and GNPS

Table S7. The precision, recall and f1 score from MS2Lipid and CANOPUS for the test queries that CANOPUS supported

Table S8. Prediction results for PC/PE co-eluted spectra

Table S9. Novel lipid subclasses characterized in this study

Supplementary Data

Supplementary Data S1: Labeled spectra and its peak metadata for 82 projects

Supplementary Data S2: Correctly labeled spectra used in the training dataset for machine learning.

Supplementary Data S3: Test queries from RIKEN LIPIDOMICS data repository

Supplementary Data S4: Peak-picking data for positive and negative ion modes from a human cohort study.

Supplementary Note

Supplementary Note S1: Mathematics theory of MCH (mod of carbon and hydrogen)-value
