# Supplementary material for: MS2Lipid: A Lipid Subclass Prediction Program Using Machine Learning and Curated Tandem Mass Spectral Data
Source: Metabolites. 2024 Nov 7;14(11):602. doi: 10.3390/metabo14110602 (PMC11596251; doi:10.3390/metabo14110602)
Supplement: Supplementary file 1 [file metabolites-14-00602-s001.zip › Supplementary Files/Supplementary Note S1.pdf]

Consider any two lipids, L_1 and L_2, that belong to the same lipid class differing only in carbon chain length and number of double bonds. The exact masses of L_1 and L_2 are denoted as M_{L1} and M_{L2} respectively. Here, the following relationship holds:

$$M_{L_1} = M_{L_2} + n_1 CH_2 + \ n_2 H_2 \ \cdots (1)$$

CH_2 and H_2 are the exact masses of CH_2 and H_2, respectively, and n_1 and n_2 are arbitrary integers. Here, define the MCH-value for a lipid L as follows:

$$MCH_L = \big((M_L \ mod \ CH_2) \ mod \ H_2\big) \ mod \ (7H_2 - \ CH_2) \ \cdots (2)$$

The MCH-values for L1 and L2 are:

$$MCH_{L_1} = \Big(\big(M_{L_1} \ mod \ CH_2\big) \ mod \ H_2 \ \Big) mod \ (\ 7H_2 - \ CH_2) \ \cdots (3)$$

$$MCH_{L_2} = \Big(\big(M_{L_2} \ mod \ CH_2\big) \ mod \ H_2 \ \Big) mod \ (\ 7H_2 - \ CH_2) \cdots (4)$$

From equation (1):

$$\begin{aligned}
MCH_{L_1} &= \Big(\Big(\big(M_{L_2} + n_1 CH_2 + n_2 H_2\big) \ mod \ CH_2\Big) mod \ H_2\Big) \ mod \ (7H_2 - \ CH_2) \\
&= \Big(\Big(\big(M_{L_2} \ mod \ CH_2 + n_2 H_2 \ mod \ CH_2\big) \ mod \ CH_2\Big) \ mod \ H_2\Big) \ mod \ (7H_2 - \ CH_2) \\
&= \Big(\big(M_{L_2} \ mod \ CH_2 + n_2 H_2 \ mod \ CH_2 - r_{CH_2} CH_2\big) \ mod \ H_2\Big) \ mod \ (7H_2 - \ CH_2) \\
&= \Big(\Big(\big(M_{L_2} \ mod \ CH_2\big) \ mod \ H_2 + \big(n_2 H_2 \ mod \ CH_2 \ - r_{CH_2} CH_2\big) \ mod \ H_2 \\
&\qquad - \ r_{H_2} H_2\Big) \ mod \ H_2\Big) \ mod \ (7H_2 - \ CH_2) \\
&= \ \Big(\big(M_{L_2} \ mod \ CH_2\big) \ mod \ H_2 + (n_2 H_2 \ mod \ CH_2 \ - r_{CH_2} CH_2) \ mod \ H_2 \\
&\qquad - \ r_{H_2} H_2\Big) \ mod \ (7H_2 - \ CH_2) \\
&= \ \Big(\Big(\big(M_{L_2} \ mod \ CH_2\big) \ mod \ H_2\Big) \ mod \ (7H_2 - \ CH_2) \\
&\qquad + \Big(\big(n_2 H_2 \ mod \ CH_2 \ - r_{CH_2} CH_2\big) \ mod \ H_2 - \ r_{H_2} H_2\Big) \ mod \ (7H_2 \\
&\qquad - \ CH_2)\Big) \ mod \ (7H_2 - \ CH_2) \\
&= \Big(MCH_{L_2} + \Big(\big(n_2 H_2 \ mod \ CH_2 \ - r_{CH_2} CH_2\big) \ mod \ H_2 - \ r_{H_2} H_2\Big)\Big) \ mod \ (7H_2 \\
&\qquad - \ CH_2) \ \cdots (5)
\end{aligned}$$

Where r_{CH2} and r_{H2} are defined as:

$$r_{CH_2} = \begin{cases} 1 \ (if \ M_{L_2} \ mod \ CH_2 + n_2 H_2 \ mod \ CH_2 > CH_2) \\ \qquad\qquad 0 \ (otherwise) \end{cases}$$

$$r_{H_2} = \begin{cases} 1\ (if\ (M_{L_2}\ mod\ CH_2)\ mod\ H_2 + (n_2H_2\ mod\ CH_2\ - r_{CH_2}CH_2)\ mod\ H_2 > H_2) \\ 0\ (otherwise) \end{cases}$$

Using integers m, k, let $n_2 = 7m + k$, where $0 \leq k < 7$.

Since $kH_2 < CH_2$, the second term of the first item in equation (5) is:

$$\left((n_2H_2\ mod\ CH_2\ - r_{CH_2}CH_2)\ mod\ H_2 - r_{H_2}H_2\right)\ mod\ (7H_2 - CH_2)$$

$$= \left((n_2H_2\ mod\ CH_2\ mod\ H_2\ - r_{CH_2}CH_2)\ mod\ H_2\ - r_{H_2}H_2\right)\ mod\ (7H_2 - CH_2)$$

$$= \left(\left((7m + k)H_2\ mod\ CH_2\ mod\ H_2\ - r_{CH_2}CH_2\right)\ mod\ H_2\ - r_{H_2}H_2\right)\ mod\ (7H_2 - CH_2) \cdots (6)$$

Since $kH_2 < CH_2$, $kH_2\ mod\ CH_2\ mod\ H_2 = 0$

Moreover, since $6H_2 < CH_2 < 7H_2$, $7H_2\ mod\ CH_2 < H_2$, thus:

$$\left((7m + k)H_2\ mod\ CH_2\right)\ mod\ H_2 = 7mH_2\ mod\ CH_2 = (7H_2 - CH_2)m\ mod\ CH_2\ \cdots (7)$$

From equation (7), equation (6) is:

$$(6) = \left(\left((7H_2 - CH_2)m\ mod\ CH_2 - r_{CH_2}CH_2\right)\ mod\ H_2\ - r_{H_2}H_2\right)\ mod\ (7H_2 - CH_2)$$

$$= \left(\left((7H_2 - CH_2)m\ mod\ CH_2 + r_{CH_2}(-CH_2\ mod\ H_2)\ mod\ H_2\right)\ mod\ H_2\ - r_{H_2}H_2\right)\ mod\ (7H_2 - CH_2)$$

$$= \left(\left((7H_2 - CH_2)m\ mod\ CH_2 + r_{CH_2}(7H_2 - CH_2)\right)\ mod\ H_2\ - r_{H_2}H_2\right)\ mod\ (7H_2 - CH_2)$$

$$= \left(\left((7H_2 - CH_2)\ (m\ mod\ CH_2) + r_{CH_2}(7H_2 - CH_2)\right)\ mod\ H_2\ - r_{H_2}H_2\right)\ mod\ (7H_2 - CH_2)$$

$$= \left(\left((r_{CH_2} + m\ mod\ CH_2)(7H_2 - CH_2)\right)\ mod\ H_2\ - r_{H_2}H_2\right)\ mod\ (7H_2 - CH_2)$$

$$= \left((r_{CH_2} + m\ mod\ CH_2)(7H_2 - CH_2)\ - r_{H_2}H_2\right)\ mod\ (7H_2 - CH_2)$$

$$= \left(-r_{H_2}H_2\right)\ mod\ (7H_2 - CH_2)$$

Thus, when $r_{H_2} = 0$, $MCH_{L_1} = MCH_{L_2}$.

Therefore, for any two lipids L_1 and L_2 belonging to this lipid class, $MCH_{L1} = MCH_{L2}$ holds.
